# Mucin 1-mediated chemo-resistance in lung cancer cells

**DOI:** 10.1038/oncsis.2015.47

**Published:** 2016-01-18

**Authors:** S Y Ham, T Kwon, Y Bak, J-H Yu, J Hong, S K Lee, D-Y Yu, D-Y Yoon

**Affiliations:** 1 Department of Bioscience and Biotechnology, Bio/Molecular Informatics Center, Konkuk University, Seoul, Republic of Korea; 2 Department of Bacteriology and Genetic, Food Research Institute (FRI), Molecular and Environmental Toxicology Center (METC), University of Wisconsin, Madison, WI, USA; 3 College of Pharmacy, Medical Research Center, Chungbuk National University, Cheongju, Republic of Korea; 4 College of Pharmacy, Seoul National University, Seoul, Republic of Korea; 5 Aging Intervention Research Center, Korea Research Institute of Bioscience and Biotechnology (KRIBB), Daejeon, Republic of Korea

## Abstract

Paclitaxel (PTX) is a commonly used drug to treat diverse cancer types. However, its treatment can generate resistance and the mechanisms of PTX-resistance in lung cancers are still unclear. We demonstrated that non-small cell lung cancers (NSCLCs) survive PTX treatment. Compared with the progenitor NSCLC A549 cells, the PTX-resistant A549 cells (A549/PTX) displayed enhanced sphere-formation ability. The proportion of the cancer stem cell marker, aldehyde dehydrogenase-positive cells, and epithelial–mesenchymal transition signaling protein levels were also elevated in A549/PTX. Importantly, the levels of oncoproteins phosphoinositide-3 kinase/Akt, mucin 1 cytoplasmic domain (MUC1-C) and β-catenin were also significantly elevated in A549/PTX. Furthermore, nuclear translocation of MUC1-C and β-catenin increased in A549/PTX. The c-SRC protein, an activator of MUC1-C, was also overexpressed in A549/PTX. These observations led to the hypothesis that enhanced expression of MUC1-C is associated with stemness and PTX resistance in NSCLCs. To test this, we knocked down or overexpressed MUC1-C in A549/PTX and found that inhibition of MUC1-C expression coupled with PTX treatment was sufficient to reduce the sphere-forming ability and survival of A549/PTX. In summary, our *in vitro* and *in vivo* studies have revealed a potential mechanism of MUC1-C-mediated PTX resistance and provided insights into a novel therapeutic measure for lung cancers.

## Introduction

Lung cancer has high incidence and mortality rates^1^ and is classified into two major histological types: small cell lung carcinoma and non-small cell lung carcinoma (NSCLC). NSCLC, which includes lung adenocarcinoma, squamous cell lung carcinoma and large-cell lung carcinoma, accounts for 85% of all lung cancer cases.^2^ Present treatment options include surgical resection, radiation therapy and chemotherapy.^3^ Specifically, chemotherapy is widely practiced in clinics for the treatment of various cancers, including lung cancer. However, conventional chemotherapeutic drugs diffuse into normal cells in an aggressive and nonspecific manner resulting in unwanted organ-related toxicity.^4^ Paclitaxel (PTX) is one of the important first-line chemotherapeutic agents used against a wide range of malignancies.^5^ It has proven efficacy against multiple cancers, including breast, ovarian, prostate and NSCLC and small cell lung cancer.^6^ Taxanes such as PTX and docetaxel are highly effective for the treatment of cancer and several other diseases. These drugs target the microtubule cytoskeleton that is important in cell division. Taxane-type resistance arises when microtubule mutations have occurred, and the development of taxane resistance limits the efficacy of this types of drugs, such as PTX.^7, 8, 9^

Properties of the small group of cancer cells called tumor-initiating or cancer stem cells (CSCs) involved in drug resistance, metastasis and relapse of cancers can significantly affect tumor therapy.^10^ In addition to microtubule mutations, CSCs can cause drug resistance. CSCs, a subpopulation of cancer cells, are stem cell-like in their ability to self-renew and differentiate asymmetrically and are thought to be a source of various cells types/lineages in the tumor.^10^ Current radiotherapy and chemotherapy kill the bulk of cancer cells but often are not able to eliminate the critical CSCs, which are protected by specific resistance mechanisms. Surviving CSCs give rise to new tumors and metastases, causing relapse of the disease. The recurrent tumors become more malignant, fast spreading and resistant to radiotherapy and previously used drugs, making the prognosis for cancer patients dismal. Thus the specific survival of CSCs could provide an explanation for many therapeutic failures and highlight new directions for the enhancement of cancer therapy.^10^ CSC levels are increased in drug-resistant cancer and may lead to cancer cell survival after chemotherapy exposure.^11^ It has been suggested that PTX-resistant cancer cells have mutated transport genes and display stemness features.^12, 13^ CSCs are also responsible for cancer recurrence. Based on these theories, further cancer therapies might focus on the elimination of CSCs.^14^

Mucin 1 (MUC1) is translated as a single polypeptide that undergoes autocleavage into N-terminal (MUC1-N) and C-terminal (MUC1-C) subunits.^15^ MUC1-N contains the highly glycosylated tandem repeats that are characteristic of the mucin family.^15^ MUC1-C is a single-pass transmembrane protein that interacts with receptor tyrosine kinases, such as early growth factor receptor (EGFR) and others.^16^ Several studies reported that MUC1-C blocking can inhibit mutant EGFR-mediated signaling in lung cancer cells.^17^ The available evidence indicates that MUC1-C promotes EGFR-mediated signaling.^18^ In this context, the MUC1-C cytoplasmic domain functions as a substrate for EGFR and c-Src phosphorylation.^18^ In turn, the MUC1-C pYEKV motif serves as a binding site for the c-Src SH2 domain.^19^ MUC1-C contains highly conserved serine and tyrosine residues. When these residues are phosphorylated, MUC1-C is cleaved from MUC1 and subsequently activates nuclear factor-κB, phosphoinositide-3 kinase (PI3K)/Akt, and β-catenin.^20, 21, 22^ MUC1-C drives epithelial–mesenchymal transition (EMT) and thereby confers stemness. Targeting MUC1-C thus reverses EMT and inhibits self-renewal in mutant K-ras NSCLC cells.^23^

Previously, we have identified the PTX-resistant NSCLC A549 cells named A549/PTX.^24^ By comparing with its progenitor, we have found that CSC populations are more frequent in A549/PTX. Our *in vitro* and *in vivo* studies have revealed that A549/PTX cells have higher levels of MUC1-C-induced stemness factors and survival signaling factors than the progenitor A549 cells. We further present data indicating that MUC1-C has a pivotal role in inducing stemness and PTX resistance in human NSCLC A549 cells. In summary, the present study provides important bases for the development of novel lung cancer therapy targeting MUC1-C to reduce chemo-resistance and stemness.

## Results

### PTX resistance is mediated via a regulator of EMT in A549 cells

**Figure 1:**
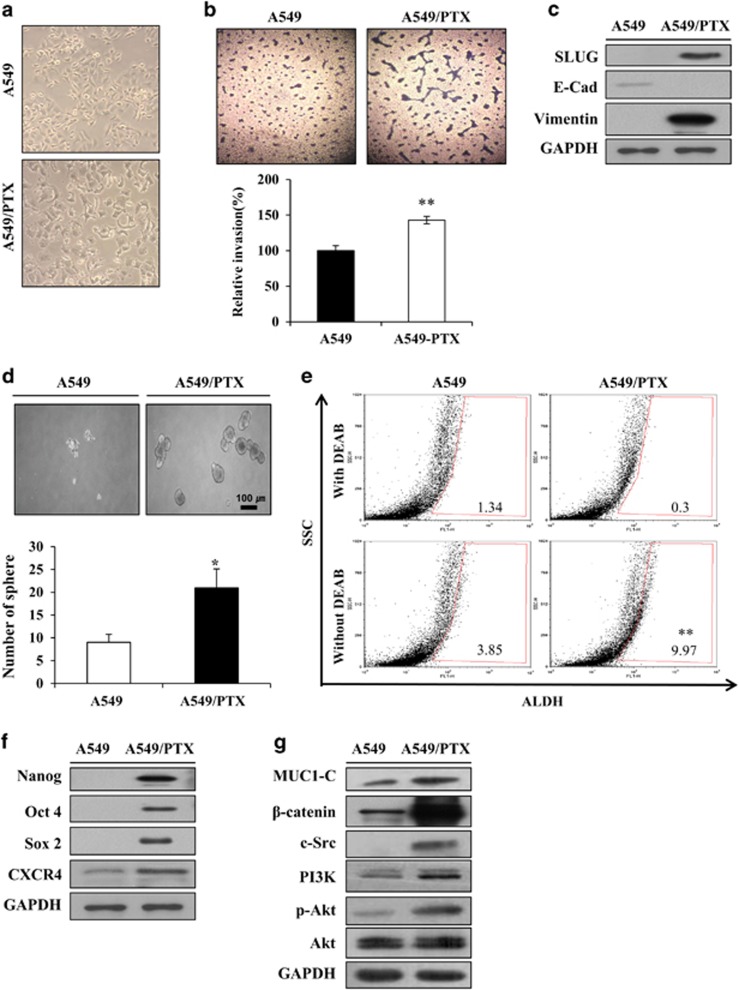
PTX resistance increases CSCs traits in A549. (**a**) Morphology of A549/PTX cells. (**b**) Invasion assay of A549 and A549/PTX cells. Images were captured at a magnification of × 200. Percentage of relative invasion in A549 and A549/PTX cells. (**c**) Expression level of EMT markers in A549 and A549/PTX cells. (**d**) Sphere formation in the A549 and A549/PTX cells cultured for 10 days (tight, spherical, non-adherent masses >100 μm in diameter; magnification, × 40). Bar represents 100 microns. Sphere formation efficiency=colonies/input cells × 100%. (**e**) FACS analysis of ALDH-positive cells in A549 and A549/PTX cells. (**f**) Expression level of stemness genes and (**g**) survival factors in A549 and A549/PTX cells. Data are the mean±s.e.m. (*n*=5) *P<0.05 and **P<0.01.

**Figure 2:**
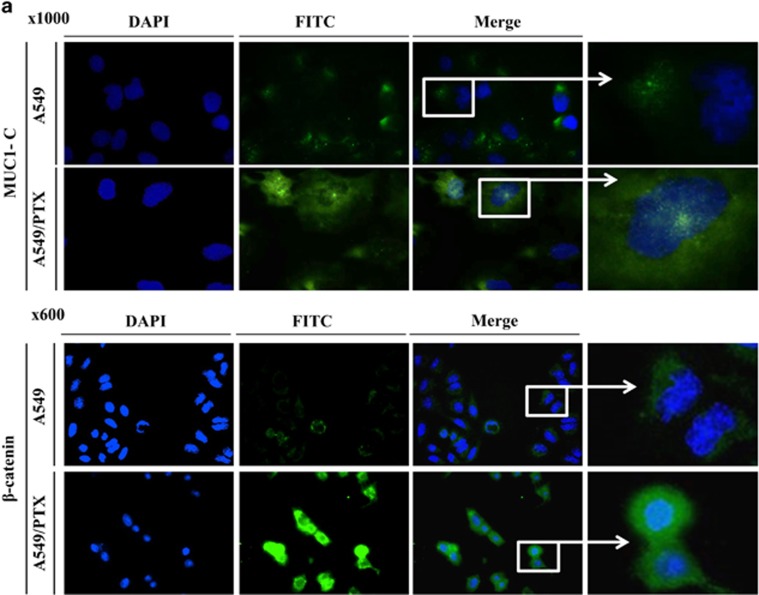
Immunofluorescence of MUC1-C and β-catenin in A549/PTX. (**a**) A549 and A549/PTX cells were fixed and assayed with immunofluorescence. DAPI nuclear staining is shown at low magnification; the boxed regions are at a higher magnification. Fluorescence images of MUC1-C (magnification, × 1000) and β-catenin (magnification, × 600).

Previous studies have reported that drug-resistant cancer cells have active stem-like features and certain characteristics.^14^ Before generating the PTX-resistant cell line, we examined a panel of lung cancer cell lines for their expression of MUC1 and β-catenin. Several lung cancer cell lines expressed high levels of MUC1-C at a basal level (Supplementary Figure S1). The A549 NSCLC cell line expressed low levels of MUC1-C compared with mucoepidermoid pulmonary carcinoma NCI-H292, bronchioalveolar carcinoma NCI-H358 cells and adenocarcinoma NCI-H1975 lines. Expression levels of β-catenin varied between different cell lines and also did not match MUC1-C expression. A549 cells significantly low expressed levels of MUC1-C and β-catenin in A549 cells compared with the other cell lines, and therefore, we selected this as a candidate NSCLC line for generating a resistance cell line to assess the roles of both proteins. To understand the changes caused by PTX resistance, we first compared the traits of A549/PTX cells and the progenitor A549 cells. As shown in Figure 1a, several morphological changes of the A549/PTX cells were evident compared with the A549 cells, which include spindle-shaped cells with an increased formation of pseudopodia. When these invasion activities were examined, PTX resistance induced both processes in A549 cells (Figure 1b). Such morphological changes and increased cell invasiveness are typical characteristics of EMT. The mesenchymal marker vimentin, epithelial marker E-cadherin and transcription factor Slug are involved in metastasis signaling.^25^ Induction of PTX resistance in A549 cells altered epithelial cell-like characteristics to mesenchymal cell-like characteristics to downregulate the expression of the epithelial marker E-cadherin, whereas upregulating the expression of the mesenchymal markers vimentin and Slug as shown in Figure 1c. This suggests that A549/PTX cells can promote invasion by inducing an EMT. The involvement of Slug in this process can be assessed by comparing Slug levels in A549/PTX and A549 cells. These led us to hypothesize that PTX resistance is mediated via a regulator of EMT in A549 cells. The importance of EMT as a driver of invasion and metastasis is being increasingly recognized. Recent studies have highlighted a link between EMT and the CSCs that initiate and maintain tumors.^26^ The sphere-forming assay is widely used to identify stem cells retrospectively based on the utility of the assay in evaluating self-renewal and differentiation at the single-cell level *in vitro*.^27^ We next investigated sphere formation in A549/PTX cells. As shown in Figure 1d, spheroid formation was seen within 10 days, indicating that A549/PTX cells have greater sphere-forming capacity and formed more spheres than parent A549 cells. It was confirmed by using aldehyde dehydrogenase (ALDH) inhibitor that the level of ALDH, a known CSC marker, was increased in A549/PTX cells. ALDH activity has been proposed to be a useful marker for CSCs, which exhibit elevated ALDH1 expression.^28^ We examined whether the ALDH-positive cell was significantly increased in A549/PTX cells compared with A549 cells (Figure 1e). A549/PTX cells possess a significant portion of cancer stem-like cells. Stemness genes (Nanog, Oct4 and Sox2) and C-X-C chemokine receptor 4 (CXCR4) have important roles in maintaining CSC signaling and stemness in drug-resistant cancer cells.^11^^,^^26^ As expected, stemness-related factors such as Nanog, Oct4, Sox2 and CXCR4 were overexpressed in A549/PTX cells (Figure 1f). Previously, other reports have demonstrated that inhibiting MUC1-C in several different ways is associated with suppression of key pathways that reside downstream of mutant K-ras and are necessary for growth and survival.^23^ When MUC1-C is activated by c-Src, it moves into the nucleus with β-catenin.^29^ We revealed that the expression levels of MUC1-C, β-catenin and c-Src were enhanced in A549/PTX cells (Figure 1g). To confirm MUC1-C- and β-catenin-expressed location, we performed immunofluorescence of MUC1-C and β-catenin in A549/PTX cells. MUC1-C was located in the nucleus of the particular foci, while MUC1-C was located in the cytosol of A549 cells. These results indicate that MUC1-C moves into the nucleus when A549 cells are resistant to PTX. A549/PTX-resistant cells expressed more β-catenin compared with A549 cells as shown Figure 1g, which transported into the nucleus in these resistant cells (Figure 2a). The results show that PTX resistance confers stemness and EMT phenotypes in A549 cells, and A549/PTX cells overexpressed EMT, stemness and survival signaling factors, including MUC1-C and β-catenin.

### A549/PTX cells exhibited proliferation and CSC self-renewal

**Figure 3:**
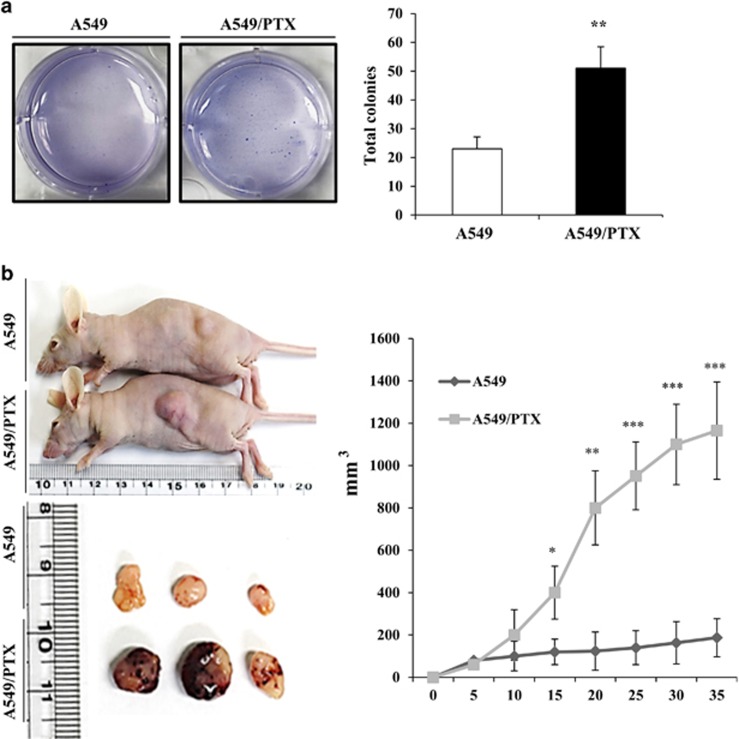
Proliferation and tumor growth of A549/PTX cells. (**a**) Soft agar assay of A549 and A549/PTX cells. (**b**) The xenograft analysis. Tumor volumes were observed for 35 days after injection of A549 (▄) and A549/PTX (♦) cells onto nude mice. Data are the mean±s.e.m. (*n*=5) *P<0.05, **P<0.01, ***P<0.001.

**Figure 4:**
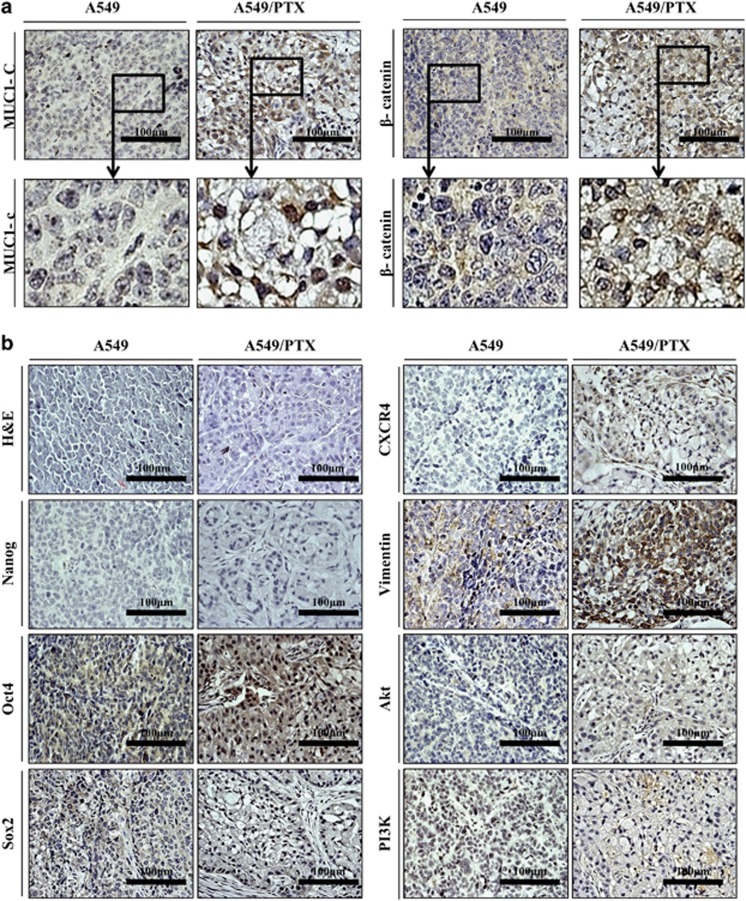
Immunohistochemical staining of A549/PTX cell tumors. (**a**) Immunochemistry images of A549 and A549/PTX tumors using respective antibodies specific to MUC1-C and β-catenin. The boxed regions are at a higher magnification. (**b**) Immunohistochemical analysis of stemness genes, EMT markers and survival factors (magnification, × 400).

The cell proliferation and tumor growth ability were examined with an *in vitro* colony-formation assay and an *in vivo* xenograft model with nude mice using A549/PTX and A549 cells. As shown in Figure 3a, colony numbers were increased using the A549/PTX cells. Therefore, this result suggests that PTX-resistant cells proliferate faster than A549 cells in the lung cancer cell line. Therefore, we injected A549 (1 × 10^5^) and A549/PTX cells (1 × 10^5^) into the flanks of 5-week-old nude mice. A549/PTX tumors exhibited more rapid growth than A549 tumors. After 35 days, the mice were killed and the two kinds of tumors were compared. We observed that the A549/PTX were significantly larger than the A549 (Figure 3b). We harvested tumors and performed immunohistochemistry to validate results from our *in vitro* assay. The expression levels of MUC1-C and β-catenin were examined in A549 and A549/PTX tumors by using immunohistochemistry. MUC1-C and β-catenin were overexpressed and translocated into the nucleus in A549/PTX (Figure 4a). Therefore, stemness proteins, in particular, oct4, EMT markers, survival factors and CXCR4, were overexpressed in A549/PTX tumors compared with A549 tumors (Figure 4b). These data indicate that A549/PTX cells exhibited greater proliferation and self-renewal abilities than A549 cells. Moreover, PTX-resistant cells overexpress the level of stemness, EMT and survival proteins.

### MUC1 regulated stemness in A549/PTX

**Figure 5:**
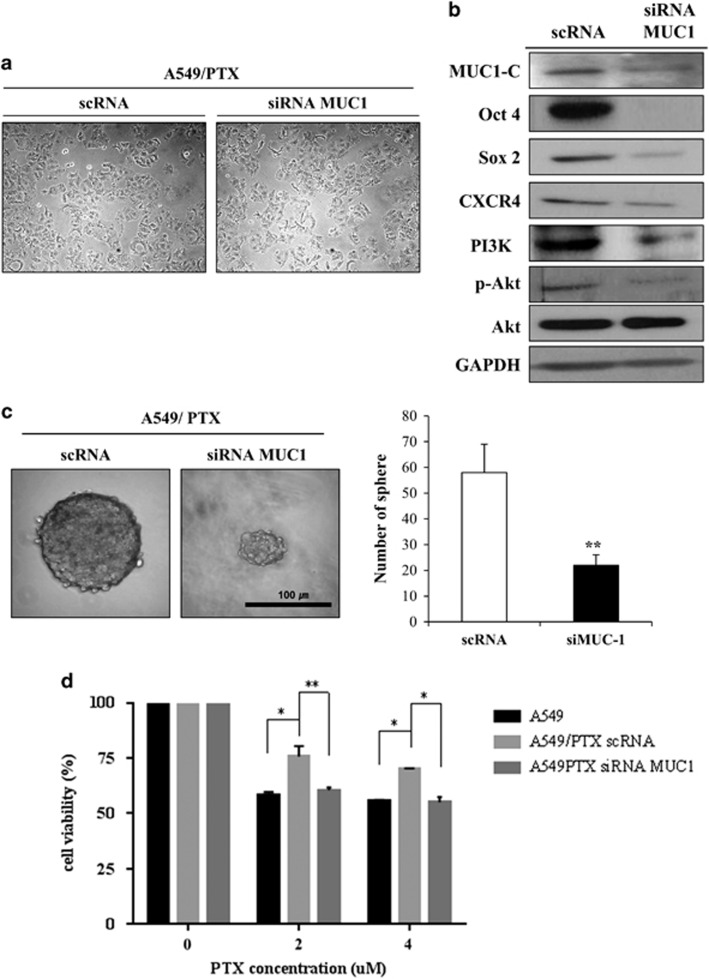
Effects of MUC-1 siRNA transfection on cell viability and sphere formation in A549/PTX cells. (**a**) Images of A549/PTX cells transfected with scRNA or MUC1 siRNA. (**b**) The expression levels of MUC1, c-Src, PI3K/Akt and Stemness-related factors in A549/PTX cells transfected with siMUC1. (**c**) Sphere formation in the A549 and A549/PTX cells cultured for 10 days (magnification, × 200). Bar represents 100 microns. (**d**) Cell viability for PTX treatment in A549 cells, A549/PTX and MUC1-silencing A549/PTX cells. Data are the mean±s.e.m. (*n*=5) *P<0.05, **P<0.01.

Overexpression of MUC1, as found in breast cancer cells, is also associated with resistance to apoptosis in response to genotoxic anticancer agents.^30^ Other reports have demonstrated that MUC1 expression is increased in breast cancer cells that form mammospheres.^31^ We tested two small interfering RNAs (siRNAs) that targeted different places of MUC1 and confirmed effective blockage of MUC1-C level in the first siRNA (Supplementary Figure S1B). In order to elucidate the differential effect of MUC1 on stemness and resistance to PTX, cell viability and sphere-forming ability were assessed after the introduction of MUC1 siRNA into A549/PTX cells. However, there was no effect of MUC1 siRNA treatment on the proliferation and morphology of A549/PTX cells (Figure 5a). To elucidate the effect of MUC1 on the expression of stemness and survival signaling pathway genes, we examined c-Src, PI3K/Akt, Oct4, Sox2 and CXCR4 expression following MUC1 siRNA treatment in A549/PTX cells. As shown in Figures 5b and c, Src expression was not altered (data not shown), whereas Akt expression was increased and PI3K/p-Akt expression was reduced in MUC1 knockout A549/PTX cells. In addition, Oct4, Sox2 and CXCR4 expression levels were decreased in the presence of MUC1 siRNA in A549/PTX cells (Figure 5b). Moreover, silencing of MUC1 resulted in the suppression of the sphere-forming ability of A549/PTX cells (Figure 5c). MUC1 silencing in A549/PTX cells furthermore reversed the PTX resistance to levels seen in A549 parental cells (Figure 5d). Taken together, these data demonstrate that MUC1 has an important role in inducing stemness and PTX resistance in A549 cells. Overall, our data support the targeting of MUC1 to reduce PTX resistance via blocking stemness features in lung cancer.

### Overexpression of MUC1 was involved in activating self-renewal, proliferating and sphere-forming ability

**Figure 6:**
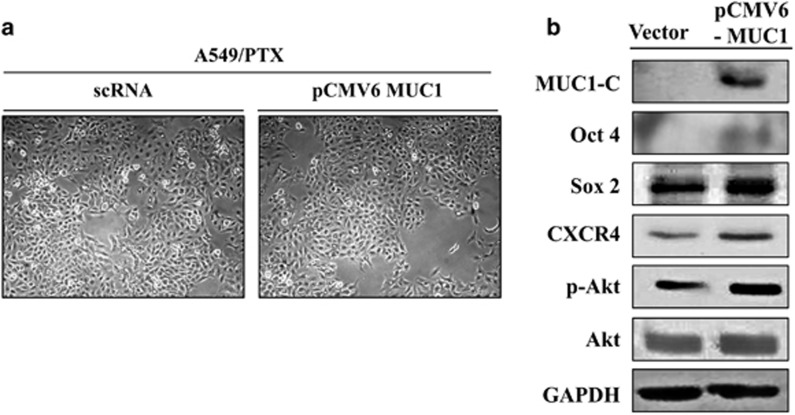
Effects of overexpressing MUC1 on stemness and PI3K signaling. (**a**) Images of A549 and MUC1-overexpressing A549 cells. (**b**) Lysates of A549 cells and MUC1-overexpressing A549 cells were immunoblotted with respective antibodies to Oct4, Sox2, CXCR4 and Akt signaling factors.

To support the relation of MUC1 and investigate self-renewal potential of NSCLC cells, we generated A549 cells overexpressing MUC1 (Figure 6a). Oct4/Sox2 and CXCR4 expression levels were increased in MUC1-overexpressing A549 cells. In addition, MUC1-overexpressing cells showed activated Akt (Figure 6b). This clearly demonstrated that MUC1 expression was involved in CSC features as well as Akt pathway.

## Discussion

Present cancer treatment options include surgical resection, radiation therapy and chemotherapy.^3^ PTX is one of the most frequently used anticancer drugs for ovarian and lung cancer patients. However, several cancers are resistant to taxan-based drugs.^32^ We investigated the role of MUC1-C in the stemness of A549/PTX cells. The A549 NSCLC line was chosen because the cells express lower levels of MUC1 protein compared with other lung cancer cells.^21, 33^

MUC1 is a transmembrane heterodimeric protein that is aberrantly expressed in NSCLCs. Over 80% of NSCLCs of the adenocarcinoma subtype express MUC1 at high levels.^34^ In addition, the overexpression of MUC1 in NSCLC is associated with poor disease-free and overall survival.^34^ GO203, an inhibitor of MUC1-C that has been designed to prevent MUC1-C nuclear translocation and oncogenic signaling, has been used for breast cancer patients in Phase I clinical trials.^15^ More recently, the role of MUC1 in the expression of stemness characteristics such as self-renewal capacity and ALDH induction in breast cancer has been evaluated.^35, 36^ MUC1 is known to contribute to the resistance to anticancer agents in some carcinomas.^30, 33^

CSC levels are increased in drug-resistant cancer and may lead to cancer cell survival after chemotherapy exposure.^10^ Our studies revealed that resistance to PTX correlates to MUC1-C expression and stemness. A549/PTX cells have a greater sphere-forming ability, a larger ALDH1-positive CSC population and higher expression levels of Nanog, Oct4, Sox2, CXCR4 and Akt compared with A549 cells. These results indicate that PTX-resistant lung cancer cell populations contain more CSCs than non-PTX-resistant lung cancer cell populations. MUC1-C translocalization into the nucleus recruits transcriptional co-activators, resulting in stemness.^37^ Immunohistochemistry and confocal immunostaining analyses also revealed that MUC1-C was translocalized into the nucleus of A549/PTX cells but not of A549 cells, suggesting that nuclear localization of MUC1 may potentially activate transcriptional co-activators. These data suggest that cells with active CSC properties might develop resistance to PTX. Xenograft tissues yielded results similar to those obtained in *in vitro* tests; specifically, the transcription factor, Oct4, was activated in A549/PTX cells. Previous studies reported that Sox2 and Oct4 are associated with sphere-forming ability and drug resistance.^38^ MUC1 have a Oct4 and Sox2 high expression levels also have been colony-forming ability.^39^ PI3K signaling is closely involved in MUC1 signaling, as PI3K can bind with MUC1-C and this binding can be disrupted by an MUC1 inhibitor. Additionally, when MUC1 is blocked, the PI3K–mammalian target of rapamycin pathway is downregulated.^21^ Furthermore, MUC1 knockdown in A549/PTX cells showed reduced PTX resistance, PI3K signaling and stemness, suggesting that an increase of CSC properties is associated with PTX resistance and MUC1-C expression in NSCLC. Moreover, MUC1-overexpressing cells showed similar phenotype with PTX-resistant cells.

In this study, we investigated whether MUC1-C has an essential role in inducing stemness and PTX resistance in human NSCLC A549 cells. Following *in vitro* and xenograft analyses, we concluded that MUC1-C is involved in stemness and PTX tolerance of cancer cells. Furthermore, stemness and survival-related factors are regulated by MUC1 expression. We studied lung cancer cells surviving PTX exposure and observed that PTX resistance and CSC features are controlled by MUC1-C. Recent cancer therapy research is focused on reducing CSCs. Our data demonstrated that MUC1-C is one of the targets to reduce PTX resistance and stemness in cancer therapy. Overall, co-treatment with an MUC1-C and an anticancer drug may have a synergistic effect in cancer patients with PTX-resistant tumors.

## Materials and methods

### Cell culture

A549 human NSCLC cells were obtained from the American Type Culture Collection (ATCC, Rockville, MD, USA). The cells were cultured in RPMI medium (Wellgene Laboratories, Daegu, Korea) supplemented with 2 mM L-glutamine and 10% fetal bovine serum (Hyclone Laboratories, Rockford, IL, USA) and incubated under humidified conditions at 37 °C with 5% CO_2_. The PTX-resistant A549 cell line (A549/PTX) was prepared and gifted by Dr Lee.^24^

### Flow cytometric analyses

The ALDEFLUOR assay (STEMCELL Technologies, Vancouver, British Columbia, Canada) was performed to isolate and characterize CSC populations in A549 and A549/PTX cells, according to the manufacturer's instructions. Briefly, 1 × 10^6^ cells were resuspended in Aldefluor assay buffer containing the ALDH substrate. As a negative control, an aliquot of Aldefluor-exposed cells was immediately quenched with a specific ALDH inhibitor, diethylaminobenzaldehyde. Following a 30-min incubation at 37 °C, the cells were washed and analyzed by FACSCalibur (Becton Dickinson, BD sciences, San Jose, CA, USA).

### Transfection of MUC1 siRNA

siRNA transfection was performed to transiently knock down the target gene. The MUC1 siRNA sequence is described by Ren *et al.*^30^ MUC1 siRNA was transfected using Lipofectamine RNAiMAX Transfection Reagent (iNtRON Biotechnology, SungNam, Korea), according to the manufacturer's instructions.

### Overexpression of MUC1

Transfection of pCMV6–MUC1 vector was performed to overexpress MUC1 (Origene, Rockville, MD, USA). pCMV6 and pCMV6-MUC1 vectors were transfected using LipofectaminTransfection Reagent (iNtRON Biotechnology), according to the manufacturer's instructions.

### Immunofluorescence

Immunofluorescence facilitates the identification of target protein localization. Cells were seeded on cover slips, fixed with acetone after 24 h and then washed with phosphate-buffered saline (PBS). The cover slips were incubated in PBS containing 0.5% respective primary antibodies specific to MUC1-C and β-catenin (1:500 with PBS) overnight at 4 °C. Fluorescein isothiocyanate-tagged secondary antibodies were then attached to primary antibodies for 1 h at room temperature. Nuclei were stained by DAPI (4,6-diamidino-2-phenylindole) staining. The cover slips were mounted on glass slides, and the cells were imaged by fluorescence microscopy (BX61-32FDIC, Olympus, Tokyo, Japan).

### A xenograft animal model

Five-week-old female BALB/c/nu/nu mice were purchased from Nara Biotech Co. Ltd. (Seoul, Korea). All experiments were approved by and performed according to the Guide for the Care and Use of Animals (Korea Research Institute of Bioscience and Biotechnology Animal Care and Use Committee, Daejeon, Korea). All mice were incubated in specific pathogen-free conditions. Tumorigenicities of A549 and A549/PTX cells were assayed by subcutaneous inoculations of 1 × 10^5^ cells resuspended in a mixed matrigel of PBS into the flanks of 5-week-old athymic BALB/c female nude mice (*n*=5 per group). The tumor size was measured using calipers (calculated volume=shortest diameter^2^ × longest diameter/2) in 5-day intervals. Five weeks after cell inoculation, the grafts were removed, photographed and fixed in 4% paraformaldehyde.

### Statistical analysis

Data are presented as the mean±s.e.m. of results from at least three independent experiments. Statistical significance was assessed using Student's *t*-test, with *P*<0.05 considered statistically significant. *P*<0.05, *P*<0.01 and *P*<0.001 are indicated in the figure legends.

## Supplementary Material

Supplementary Figure 1

Supplementary Informations
