# Supplementary figures and images for: Mucin 1-mediated chemo-resistance in lung cancer cells
Source: Oncogenesis. 2016 Jan 18;5(1):e185–. doi: 10.1038/oncsis.2015.47 (PMC4728677; doi:10.1038/oncsis.2015.47)

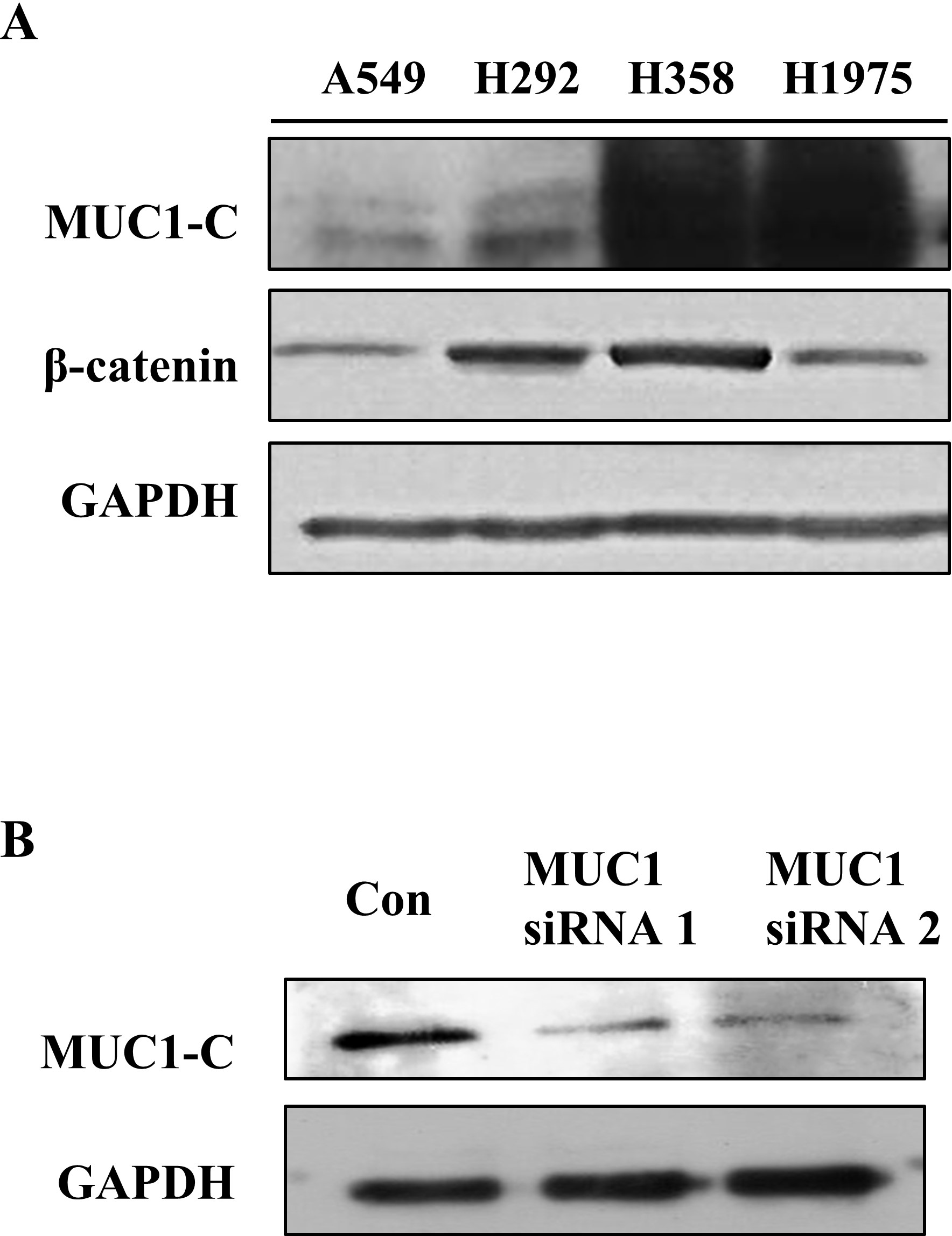

Supplement: Supplementary Figure 1 [file oncsis201547x1.tif]
