# Supplementary material for: Mucin 1-mediated chemo-resistance in lung cancer cells
Source: Oncogenesis. 2016 Jan 18;5(1):e185–. doi: 10.1038/oncsis.2015.47 (PMC4728677; doi:10.1038/oncsis.2015.47)
Supplement: Supplementary Informations [file oncsis201547x2.doc]

**Supplementary information**

**Mucin 1-mediated chemo-resistance in lung cancer cells**

Sun Young Ham^1,6^#, Taeho Kwon^1^#, Yesol Bak^1^, Jae-Hyuk Yu^2^, Jintae Hong^3^, Sang Kook Lee^4^, Dae-Yeul Yu^5^**,** and Do-Young Yoon^1^*****

^1^Department of Bioscience and Biotechnology, Bio/Molecular Informatics Center, Konkuk University, Neungdong-ro 120, Gwangjin-gu, Seoul 143-701, Republic of Korea.

^2^Departments of Bacteriology and Genetics, Food Research Institute (FRI), Molecular and Environmental Toxicology Center (METC), University of Wisconsin, Madison WI, USA .

^3^College of Pharmacy, Medical Research Center, Chungbuk National University, 12 Gashin-dong, Heungduk-gu, Cheongju, Chungbuk, 361-463, Republic of Korea.

^4^College of Pharmacy, Seoul National University, San 56-1, Sillim-dong, Gwanak-gu, Seoul 151-742, Republic of Korea.

^5^Aging Intervention Research Center, Korea Research Institute of Bioscience and Biotechnology (KRIBB), Gwahak-ro 125, Yuseong-gu, Daejeon, 305-333, Republic of Korea.

^6^Current address: Tumour Microenvironment Laboratory, QIMR Berghofer, 300 Herston Rd, Herston QLD 4006, Australia

# **Ham SY** and **Kwon T** equally contributed to this research.

**Subtitle:** MUC1-c is a target for cancer therapy.

*** Corresponding authors:**

Correspondence: **Do-Young Yoon**

E-mail: [ydy4218@konkuk.ac.kr](mailto:ydy4218@konkuk.ac.kr) , Phone: 82-2-444-4218. Fax: 82-2-444-4218.

**1. Supplementary Methods: 2-3 page**

**2. Supplementary Figure Legend: 4 page**

**1. Supplementary Methods**

**Supplementary Method S1. Western blot analysis**

To identify the protein expression levels, A549 and A549/PTX cells were seeded on 6-well plates^1^. Harvested cells were lysed in radioimmunoprecipitation assay buffer (0.1 % sodium dodecyl sulfate [SDS], 0.1 % sodium deoxycholate, 1 % Triton X-100, 1 mM EDTA, 0.5 mM ETDA, 140 mM NaCl, and 10 mM Tris-HCl, pH 8.0) containing phosphate and protease inhibitors. The cell lysate was centrifuged at maximum speed for 30 min at 4°C, and the resulting supernatant was quantified. Component proteins were separated by SDS-PAGE and transferred to polyvinylidene difluoride membranes. The membranes were blocked by incubation in 5% non-fat milk solution in Tris-buffered saline containing Tween-20 (TBST: 2.7 mM NaCl, 53.65 mM KCl, 1 M Tris-HCl, pH 7.4, 0.1 % Tween-20) for 1 h at room temperature. After blocking, the membranes were incubated in 1% milk solution containing the primary antibody in TBST for 2–4 h. After three consecutive washes in TBST, the membranes were incubated with the secondary antibodies (horseradish peroxidase [HRP]-conjugated α-rabbit or α-mouse IgG) for 1 h at room temperature. After washing the membranes four times, the signal was visualized using the Westzol plus Western Blot Detection System (iNtRON Biotechnology, SungNam, Korea).^1^

**Supplementary Method S2. Immunohistochemistry**

Immunohistochemistry analysis was performed according to the protocol provided by the antibody manufacturer.^2^ Briefly, tumor tissues were fixed with formalin and embedded with paraffin. The paraffin tissue sections were removed using xylene, hydrated, and exposed to a heat-induced epitope retrieval buffer (sodium citrate buffer, pH 6.0) by using a high-pressure cooker for 5 min. Samples were treated with 1% H_2_O_2_ diluted with methanol to block endogenous peroxidase activity and incubated on 2% BSA for 1 h. The primary antibodies specific to Oct4, Sox2, CXCR4, vimentin, PI3K, Akt, MUC1-c and β-catenin (1:200~1:500 with 5% BSA) were incubated at 4°C overnight. The sections were washed three times and incubated with a secondary antibody for 1 h at room temperature. The tissue sections were prepared using a DAB detection kit (Vector, Burlingame, CA, USA) and stained by hematoxylin (Sigma-Aldrich, MO, USA). Finally, the samples were dehydrated with graded strengths of alcohol, cleared in xylene, mounted, and covered by a cover slip for microscopic observation (400×).^2^

**Supplementary Method S3. Hematoxylin and eosin (H&E) staining**

H&E staining was performed according to the protocol provided by the H&E solution manufacturer. Briefly, the paraffin-embedded sample was subjected to changes of xylene, alcohol, and water to remove paraffin and hydrate the tissue. After hydration, the slide-mounted samples were stained by hematoxylin and eosin Y and rapidly washed with water several times. The tissue samples were dehydrated with graded strengths of alcohols, cleared in xylene, and finally covered with a cover slip using a mounting medium. The samples were then observed under a microscope (200×).^2^

**Supplementary Method S4. Soft agar colony assays**

The cells were collected at the exponential phase of growth, digested, and blown into single-cell suspensions. ^1^, ^2^ The suspensions were diluted and inoculated into six-well plates at densities of 3×10^3^ cells, with three duplicate wells set up for each cell density. The six-well plates were incubated at 37°C in 5% CO_2_ for 2 weeks. Once macroscopically visible clones appeared, the culture was terminated; the cells were rinsed, fixed, and subjected to Giemsa staining (Sigma-Aldrich, MO, USA). The clones were counted, and the cloning efficiency was calculated according to the following formula: cloning efficiency (%) = number of clones / number of inoculations × 100%.^1^, ^2^

**References**

1 Kwon T, Rho JK, Lee JC, Park YH, Shin HJ, Cho S *et al*. An important role for peroxiredoxin II in survival of A549 lung cancer cells resistant to gefitinib. Exp Mol Med 2015; **47**: e165.

2 Park YH, Kim SU, Kwon TH, Kim JM, Song IS, Shin HJ *et al*. Peroxiredoxin II promotes hepatic tumorigenesis through cooperation with Ras/Forkhead box M1 signaling pathway. Oncogene 2015.

**2. Supplementary Figure Legend**

**Supplementary Figure 1. Expression of MUC1 in lung cancer cell lines.** (A) Expression of MUC1 and β-catenin in lung cancer cell lines. (B) A549/PTX cells transfected with Con (scRNA) or MUC1 siRNA 1 and siRNA 2
